# Severe Abdominal Pain

**Published:** 1903-06-20

**Authors:** J. M. Fortescue-Brickdale

**Affiliations:** Registrar and Pathologist to the Children's Hospital, Bristol


### Hospital Clinics and Medical Progress.

### SEVERE ABDOMINAL PAIN.

By J. M. Fortescue-Brickdale, M.A., M.D. Oxon., Registrar and Pathologist to the Children's Hospital, Bristol.

(*Concluded from page* 184.)

5. *Physical Signs in the Abdomen.*—The careful examination of the abdomen is above all things imperative. In cases of perforation the abdomen is generally swollen and tympanitic, subsequently becoming board-like and retracted as definite peritonitis sets in. Rarely, however, the abdomen in perforated gastric ulcer is retracted from the first owing to spasm of the muscular walls. An important indication of severe disease is the absence of respiratory movements in the abdominal muscles; it generally denotes the rupture of a viscus, though it is also observed in diaphragmatic pleurisy. Tympanites is not a distinctive symptom; in obstruction of the bowel high up it is usually not well marked. It should be remembered that absence of liver dulness cannot be taken as indicating the presence of gas in the peritoneal cavity with any degree of certainty. In biliary colic and in appendicular disease there may be local increase of resistance, or even a definite tumour. In pyosalpinx and in ectopic pregnancy (in certain cases) a tumour may also be felt. In twisted ovarian pedicle the tumour will possibly be found. Acute hæmorrhagic pancreatitis has been diagnosed correctly by the discovery of a tender swelling along the region of the pancreas. Gall-stones and renal calculi have been felt *in situ* and even grated on one another, but this is not usually possible. McBurney's spot is of little diagnostic value in appendicitis. Intestinal peristalsis may be observed in simple colic, and in obstruction of the gut.

6. *Physical Signs Outside the Abdomen.*—In the general examination of the patient particular attention should be paid to certain points. The condition of the knee-jerks and pupil reflexes should be observed in order to exclude tabes dorsalis; the "blue line" and wrist drop of lead poisoning should be looked for; and the signs of pregnancy which may be present in rupture of an extra-uterine fœtation should be sought. In cases of renal colic retraction of the testicle may be observed, but more frequently in boys than in adults; moreover, it also occurs sometimes in appendicular colic. In apparent cases of appendicular disease in women a rectal or vaginal examination should be made to determine the absence of enlargement of the fallopian tube. The sites of a possible hernia should also be examined in all cases. Jaundice should be looked for, and an examination of the urine made for blood, etc. (renal calculus).

7. *Constipation and Diarrhœa.*—If the latter is really present intestinal obstruction is of course excluded. It is strongly suggestive of poisoning, especially in connection with vomiting. Constipation is common in many of the conditions under discussion, though not invariable in any, as even in obstruction of the intestine a little fæcal matter may be passed from below the obstruction.

8. *History.*—This may give an important clue to the cause of the condition; on the other hand it may be completely useless. For instance, perforation may be the first symptom of gastric ulcer, and jaundice is a fairly common malady which might accidentally precede any abdominal condition. Positive indications, however, are not infrequent. A history of dyspeptic attacks, with or without hæmatemesis, points to gastric ulcer; melæna, if a history of this be obtained, may indicate duodenal ulcer, but it is a symptom which if not severe is often overlooked by the patient. The possibility of irritant poisoning should be inquired into, and of exposure to lead, which is the most frequent cause of severe intestinal colic in adults. A previous history of biliary or renal calculi, of the passage of gravel or blood in the urine; pelvic troubles, such as profuse, painful, irregular menstruation, or of an apparent pregnancy, should be inquired after. Recently attention has been drawn to the occurrence of symptoms resembling acute appendicitis in cases of lead poisoning; this should be remembered, though it has been suggested that the latter condition may in some instances be causally connected with the former. A history of chronic peritonitis, or conditions which might have produced it, would suggest strangulation or obstruction of the gut.

It will be observed that little has hitherto been said of the symptoms which accompany two conditions, viz., ruptured abdominal aneurysm and acute hæmorrhagic pancreatitis. The first of these is rare and generally immediately fatal, but would of course be associated, should time allow, with symptoms of the most intense peritonism. Rupture in ectopic pregnancy with severe hæmorrhage will simulate this condition most closely, and is in women a far more probable diagnosis. The second condition resembles in its symptoms acute intestinal obstruction or perforated gastric ulcer, and has usually been diagnosed as one or other of these; the mistake, however, is not important, as operation is the only course which affords any hope of prolonging life.

In cases of severe abdominal pain which first come under observation at a later stage, the diagnosis will as a rule be easier; at any rate it will be more possible to say whether surgical interference is necessary or not. The dry tongue, extreme thirst, persistent vomiting associated perhaps with constipation, a rigid, very tender abdomen, gradually becoming distended and tympanitic and the well marked "facies hippocratica" demonstrate with certainty the grave nature of the case. An apparent change for the better may, however, occur after the establishment of definite peritonitis, but this should not give rise to error if a careful history be obtained. Peritonitis may, of course, arise from other conditions than those enumerated above; the history will often be of value in such cases, and moreover an operation, if decided upon, will depend for its success on its power of dealing with the peritonitis as much as on the possible cause of that condition.

It may be as well, in conclusion, to suggest that in all cases in which an operation is decided on, in addition to the notes of the condition the diagnosis arrived at should be written down; for such is the frailty of our mortal nature that we are all apt to be wise after the event even to the extent of forgetting the carefully-considered judgment we formulated before it.

